# Outcomes of 23- and 24-weeks gestation infants in Wellington, New Zealand: A single centre experience

**DOI:** 10.1038/s41598-017-12911-5

**Published:** 2017-10-06

**Authors:** Mary Judith Berry, Maria Saito-Benz, Clint Gray, Rebecca Maree Dyson, Paula Dellabarca, Stefan Ebmeier, David Foley, Dawn Elizabeth Elder, Vaughan Francis Richardson

**Affiliations:** 1 Department of Paediatrics & Child Health, University of Otago, Wellington, New Zealand; 2 Neonatal Intensive Care Unit, Wellington Regional Hospital, Wellington, New Zealand; 3 Graduate School of Medicine and Illawarra Health and Medical Research Institute, University of Wollongong, New South Wales, Australia; 4 The Medical Research Institute of New Zealand, Wellington, New Zealand; 5 Department of Microbiology, Wellington Regional Hospital, Wellington, New Zealand

## Abstract

Optimal perinatal care of infants born less than 24 weeks gestation remains contentious due to uncertainty about the long-term neurodevelopment of resuscitated infants. Our aim was to determine the short-term mortality and major morbidity outcomes from a cohort of inborn infants born at 23 and 24 weeks gestation and to assess if these parameters differed significantly between infants born at 23 vs. 24 weeks gestation. We report survival rates at 2-year follow-up of 22/38 (58%) at 23 weeks gestation and 36/60 (60%) at 24 weeks gestation. Neuroanatomical injury at the time of discharge (IVH ≥ Grade 3 and/or PVL) occurred in in 3/23 (13%) and 1/40 (3%) of surviving 23 and 24 weeks gestation infants respectively. Rates of disability at 2 years corrected postnatal age were not different between infants born at 23 and 24 weeks gestation. We show evidence that with maximal perinatal care in a tertiary setting it is possible to achieve comparable rates of survival free of significant neuroanatomical injury or severe disability at age 2 in infants born at 23-week and 24-weeks gestation.

## Introduction

Advances in perinatal medicine have led to a dramatic improvement in overall survival rates for extremely preterm infants^1–3^. As technological capacity increases, the need to define the threshold of infant viability and agree on the ethical boundaries of neonatal intensive care becomes more urgent^4^. Despite improved overall survival rates the burden of neurocognitive morbidity associated with extreme prematurity remains high^5–7^. However, outcomes in previously published cohort studies reflect heterogeneous patient groups combining in-born and out-born infants and those pooled from several institutions where practice variation may influence outcomes^1,7–9^. Additionally, most outcome data focus on neurodevelopmental morbidity which, whilst important, does not necessarily reflect the overall wellbeing of the child.

It remains extremely difficult to accurately predict the clinical course of an individual infant born in the periviable period of 23 weeks gestation^10,11^. Clinicians are therefore faced with the dilemma as to whether it is ethical to initiate or withhold resuscitation when the outcomes are so uncertain^12^. Professional advisory bodies^13–17^ recognise this dilemma and recommend that discussion between health professionals and the infants’ family must underpin any clinical decision to withhold or instigate resuscitation of the periviable infant^18^. Ideally this discussion should be informed by clear short- and long-term morbidity and mortality data that is pertinent to the local tertiary institution.

In the current study we sought to address, in a population of 23 and 24 weeks gestation infants, rates of death and major morbidity at time of discharge home, and rates of disability at 2 years corrected postnatal age. The secondary aim was to assess whether the above parameters differed significantly between infants born at 23 or 24 weeks gestation.

We report our primary outcome of survival at 2 years corrected postnatal age free of moderate or severe disability. Our secondary outcomes include 1: survival to (i) NICU admission, (ii) discharge home, (iii) 2 years corrected postnatal age; 2: Maternal and neonatal wellbeing at the time of birth; 3: Major neonatal morbidity.

## Methods

### Study population

Cases were identified from the birth registration of infants born between 23^+0^ and 24^+6^ weeks of gestation in Wellington Regional Hospital, between 1^st^ January 2003 and 31^st^ December 2012. Cases were retrieved from our institutional database and clinical details cross-checked against the paper clinical record. Gestational age was defined by first trimester dating ultrasound, or by date of last menstrual period if no early ultrasound scan was available. Infants were excluded if out-born, the parents declined resuscitation, or significant congenital anomalies were present. Seven infants were excluded due to congenital abnormalities including major cardiac and abdominal wall anomalies. Resuscitation was not initiated in 11 infants due to wishes of parents in agreement with the medical team. Key data were extracted including decisions to initiate or withhold newborn resuscitation, the infants’ medical course, health status at the time of discharge home and during follow-up assessments at 2–3 years corrected postnatal age.

### Maternal characteristics

Maternal age, ethnicity and health status were obtained from patient records. Chorioamnionitis was determined by the attending obstetrician and confirmed following placental histology. Prolonged rupture of membranes was defined as rupture of membranes >24 hrs before delivery. Pre-eclampsia was defined by the obstetricians’ clinical interpretation of maternal symptoms, blood pressure, blood and urinary tests. Incomplete course of steroid was defined as one dose of betamethasone given <12 hours prior to delivery. Complete course of steroid was defined as two doses of betamethasone given 12 hours apart with the second dose <7 days prior to delivery. Mode of delivery and its indication were obtained from the maternal delivery record.

### Neonatal characteristics

Birth weight was measured in the NICU using a portable scale with accuracy of +/−2 grams (Tanita Corporation, Japan) or an in-built incubator scale with accuracy of +/−10 grams (Giraffe OmniBed, GE Healthcare, UK). Birth weight centile was derived from the New Zealand – World Health Organisation growth charts (Ministry of Health, New Zealand). Neonatal sepsis was defined by presence of a positive blood, urine or CSF culture within 48 hours of sample collection. Presence of patent ductus arteriosus (PDA) was determined by an echocardiogram performed within 24hrs of birth for all infants, and in this cohort prophylactic indomethacin (ibuprofen was substituted briefly during the study period due to a national shortage of indomethacin) was given routinely if a PDA was present unless a contraindication was identified (e.g., significant thrombocytopaenia; gastrointestinal perforation or bleeding). Diagnosis of necrotising enterocolitis (NEC) was based on the Bell classification and includes stages I to III. It is customary practice in our Unit to complete 7 days of intravenous triple antibiotics (amoxycillin, gentamycin and metronidazole) and nil by mouth for infants with NEC (Bell stage I-III). Therefore, all cases significant enough to warrant treatment have been included in subsequent analysis as a measure of potentially serious gastrointestinal morbidity. Presence of intraventricular haemorrhage (IVH) and/or periventricular leucomalacia (PVL) was determined by serial cranial ultrasonography performed and interpreted by paediatric radiologists. Reported grade of IVH/PVL (Papile classification) reflects the most significant abnormality found at any time during the infants’ admission. Diagnosis of bronchopulmonary dysplasia (BPD) was made if supplementary oxygen was required at 28 days of life, and was defined as moderate if infants required <30% oxygen at 36 weeks corrected postnatal age and severe if infants required >30% oxygen at 36 weeks corrected postnatal age. Presence of retinopathy of prematurity (ROP) and need for laser therapy was determined by serial paediatric ophthalmology examination commenced between 30 and 31 weeks corrected postnatal age. Of note, in our Unit, administration of exogenous surfactant to infants born at 23 and 24 weeks gestation was universal practice; all infants therefore received at least one dose in the newborn period. Similarly, administration of probiotics only commenced in our Unit after 2013; none of the infants included in this cohort therefore received probiotic treatment.

### Definitions of moderate to severe disabilities

Moderate to severe disabilities in this study are defined broadly to reflect neurodevelopmental disability as well as respiratory, gastrointestinal and renal complications of prematurity as per the ‘classification of health status at 2 years as a perinatal outcome’ published in 2008 by the British Association of Perinatal Medicine and Royal College of Paediatrics and Child Health Working Group^19^. This allows validated reporting of morbidity outcomes in children unable to access a standardised post-discharge developmental surveillance program. Many of the children in this cohort were domiciled in rural communities where follow-up is with a Paediatrician but may not include specialist neurodevelopmental assessment using Bayley or Griffith neurodevelopmental scales. Health outcomes were obtained by review of clinical records at 2 years corrected postnatal age. All records were independently assessed by at least 2 paediatric-trained investigators and reconciled by a third senior, paediatric-trained investigator if necessary. Wellbeing was assessed in domains of: neurodevelopmental (including motor, cognitive, hearing, speech & language, vision), respiratory, renal and gastrointestinal. One or more impairment in any ‘severe disability’ domain was classified as the child having severe disability. Similarly, one or more impairment in the ‘moderate’ disability domain was required for classification as moderately disabled (Table 1).

**Table 1:** Summary of wellbeing at 2 years corrected postnatal age.

Domain	Severe neurodevelopmental disability	Moderate neurodevelopmental disability
Motor	CP with GMFCS ≥ 3	CP with GMFCS 2
Cognitive	Score ≤−3SD below norm on formal testing Or Paediatrician assessment of severe disability	Score between -3SD and -2SD below norm on formal testing Or Paediatrician assessment of reduced cognitive function
Hearing	No useful hearing even with aids	Hearing loss corrected with aids
Speech & language	No words	<5 words
Vision	No useful vision	Moderately reduced vision in both eyes, or unilateral blindness with good vision in contralateral eye
Gastrointestinal	TPN, NG, or PEG requirement	Special diet or stoma
Renal	Dialysis or awaiting renal transplant	Renal impairment needing treatment or special diet

GMFCS: Gross motor function classification scales. TPN: total parenteral nutrition. NG: nasogastric tube feeding. PEG: percutaneous entero-gastrostomy.

### Statistical analysis

Stata 13 for MacOSX (StataCorp LP, Texas, USA) was used for statistical analyses. χ2 analysis was used to compare outcomes between those born at 23 vs. 24 weeks gestation. Associations between maternal characteristics and infant outcome were explored using multivariate regression analysis. The level of statistical significance for all analyses was set at p ≤ 0.05 using two-tailed comparisons. Data are presented as the mean ± SEM or median (range) as indicated in the text. Percentage survival rate was determined using the number of live born infants at each gestational age as the denominator for all outcomes.

### Ethical approval

Institutional ethics approval to review the health records was prospectively obtained from the Wellington Hospital Clinical Audit and Research Committee. All health records were anonymised and de-identified prior to analysis. All methods and patient information were performed in accordance with the relevant guidelines and regulations outlined by the Wellington Hospital Clinical Audit and Research Committee.

## Results

**Figure 1:**
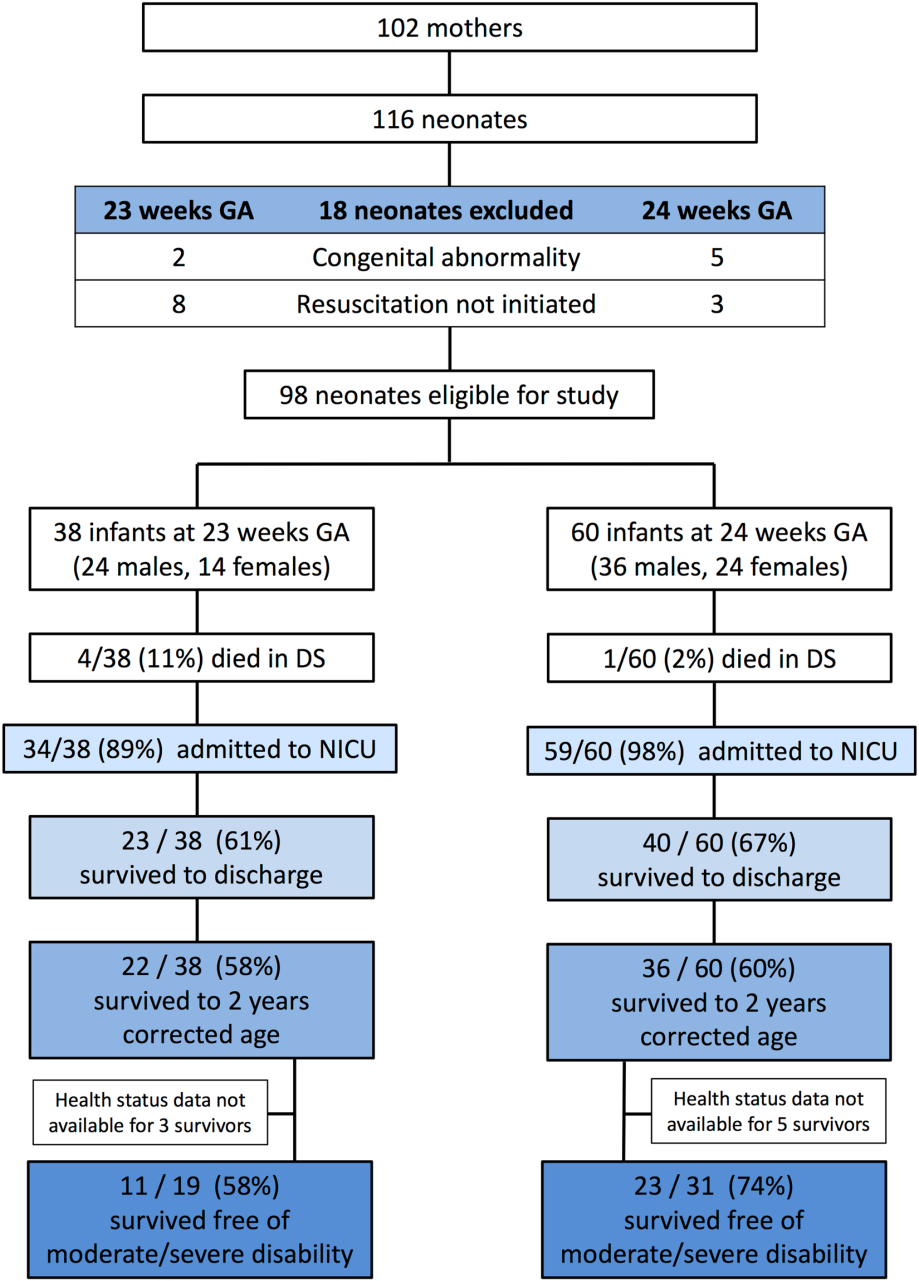
Overview of the study cohort. GA indicates gestational age. DS indicates delivery suite. All percentages are calculated with the number of live births as the denominator.

116 infants were born at 23 and 24 weeks gestation from 102 mothers. Of 98 infants eligible for study inclusion, 38 were born at 23^+0−6^ gestation and 60 were born at 24^+0−6^ gestation. There were more males (n = 60) than females (n = 38) (Fig. 1). 34/38 infants of the 23-week cohort and 59/60 infants of the 24-week cohort were admitted to NICU (Fig. 1). Resuscitation was unsuccessful in four infants born at 23 weeks gestation (all male, all had received a partial course of antenatal corticosteroid, median birth weight 600 g (560–780 g)) and one born at 24 weeks gestation (male, birth weight of 500 g and had received a full course of antenatal corticosteroid).

### Maternal characteristics

Following exclusion of neonates according to the previously defined exclusion criteria, 90 mothers were included in the analysis. There was no maternal difference between the 23-week and 24-week cohort in any perinatal variables (Table 2). For purposes of reporting perinatal outcomes, mothers of twins are only included once, with the exception of three mothers (1 with twins born at 23 weeks, and 2 with twins born at 24 weeks). As these mothers had one twin who survived, and one non-surviving twin, these mothers are included in the final numbers for both “survivors” and “non-survivors”.

**Table 2:** Maternal characteristics of the study group.

Characteristic/Morbidity	23 weeks GA (n = 36)	24 weeks GA (n = 54)
Maternal age (years) *Survivors (n* = *57)*	30 (15–40)	31 (17–39)
*Non-Survivors (n* = *33)*	30 (17–40)	33 (18–39)
Multiple pregnancy *Survivors (n* = *57)*	4 (19%)	7 (19%)
*Non-Survivors (n* = *33)*	1 (7%)	4 (22%)
Pre-eclampsia *Survivors (n* = *57)*	3 (14%)	3 (8%)
*Non-Survivors (n* = *32)*	0	5 (29%)
Chorioamnionitis *Survivors (n* = *47)*	6 (40%)	17 (53%)
*Non-Survivors (n* = *27)*	7 (58%)	7 (47%)
Rupture of membranes >24 h *Survivors (n* = *56)*	7 (33%)	12 (34%)
*Non-Survivors (n* = *33)*	6 (40%)	5 (28%)
Antepartum haemorrhage *Survivors (n* = *57)*	2 (10%)	6 (17%)
*Non-Survivors (n* = *33)*	4 (27%)	1 (6%)
Vaginal breech delivery^†^ *Survivors (n* = *26)*	3 (23%)	6 (46%)
*Non-Survivors (n* = *21)*	6 (55%)	3 (30%)
Caesarean section Delivery *Survivors (n* = *57)*	8 (38%)	22 (61%)
*Non-Survivors (n* = *33)*	4 (27%)	8 (44%)
Lower segment caesarean section^‡^ *Survivors (n* = *31)*	7 (88%)	21 (91%)
*Non-Survivors (n* = *12)*	3 (75%)	6 (75%)
Fetal indication for operative delivery *Survivors (n* = *31)*	8 (100%)	20 (90%)
*Non-Survivors (n* = *12)*	4 (100%)	5 (63%)
Maternal smoking *Survivors (n* = *52)*	4 (20%)	14 (44%)
*Non-Survivors (n* = *30)*	2 (14%)	4 (25%)

Data presented as median (range) for continuous variables or n (%) for categorical variables. % listed is calculated from number of infants/mothers with information available, not total participants (98 infants; 63 survivors, 35 non-survivors). ^†^Calculated from vaginal deliveries only (caesarean section deliveries excluded from count). ^‡^Lower segment caesarean rate calculated as a proportion of the total caesarean rate.

### Post-admission Neonatal course

Twenty-three infants in the 23-week cohort (61%) and 40 infants in the 24-week cohort (67%) survived to discharge home (Fig. 1). Birth weight was not significantly different between 23 & 24-week infants. However, within the 24-week cohort, birth weight was significantly higher in survivors when compared to non-survivors (median, range: 678, 535–880 *vs*. 630, 430–800, p = 0.05). Apgar score at 5 minutes was significantly lower in 23-week infants when compared to 24-week infants (median, range: 7, 2–9 *vs*. 9, 3–10 p < 0.01) (Table 3). Culture positive sepsis (early & late) was common in both age cohorts. Of the survivors, those born at 23-weeks required a longer total period of mechanical ventilation during the entire duration of admission than those born at 24-weeks gestation (median, range: 28, 4–68 days *vs*. 14, 2–60 days, p = 0.002). No difference in rates of NEC, ROP or any other medical or surgical complications were observed between or with groups (Table 3). Indomethacin/ibuprofen to treat a PDA was given to most infants at 23 and 24 weeks gestation (Table 3). Further surgical ligation of the PDA was required in four infants born at 23 weeks gestation of whom two survived, and five infants born at 24 weeks gestation of whom two survived.

**Table 3:** Characteristics and outcomes of infants admitted to NICU.

	23 weeks GA (n = 34)	24 weeks GA (n = 59)	p-value
Birth weight (g) *Survivors (n* = *63)*	635 (475–800)	678 (535–880)	0.03
*Non- Survivors (n* = *30)*	630 (525–740)	630 (430–800)*	NS
Birth weight <10^th^ percentile *Survivors (n* = *63)*	3 (13%)	5 (13%)	NS
*Non- Survivors (n* = *30)*	1 (9%)	6 (32%)	NS
Any antenatal corticosteroids *Survivors (n* = *63)*	20 (91%)	39 (100%)	NS
*Non- Survivors (n* = *30)*	11 (100%)	18 (95%)	NS
Completed antenatal corticosteroids *Survivors (n* = *63)*	12 (60%)	12 (67%)	NS
*Non- Survivors (n* = *30)*	6 (55%)	15 (83%)	NS
Cord pH *Survivors (n* = *63)*	7.3 (6.9–7.5)	7.4 (7.0–7.4)	NS
*Non- Survivors (n* = *30)*	7.3 (6.8–7.4)	7.3 (7.1-7.4)	NS
Apgar score at 5 minutes *Survivors (n* = *63)*	7 (2–9)	9 (3–10)	0.009
*Non- Survivors (n* = *30)*	8 (3–10)	9 (4–10)	NS
Inotrope requirement *Survivors (n* = *63)*	12 (52%)	18 (45%)	NS
*Non- Survivors (n* = *30)*	7 (64%)	9 (53%)	NS
PDA treated with indomethacin/ibuprofen *Survivors (n* = *63)*	23 (100%)	33 (83%)	NS
*Non- Survivors (n* = *30)*	10 (91%)	14 (78%)*	NS
Proven systemic infection *Survivors (n* = *63)*	20 (87%)	26 (65%)	NS
*Non- Survivors (n* = *30)*	7 (64%)	7 (37%)*	NS
Necrotising enterocolitis (Bell’s I–III) *Survivors (n* = *63)*	9 (39%)	12 (30%)	NS
*Non- Survivors (n* = *30)*	3 (27%)	7 (54%)	NS
Days on mechanical ventilation (total) *Survivors (n* = *63)*	28 (4–68)	14 (2–60)	0.002
*Non- Survivors (n* = *30)*	6 (1–85)	14 (1–62)	NS
Home oxygen *Survivors (n* = *63)*	11 (48%)	14 (36%)	NS
*Non- Survivors (n* = *30)*	n/a	n/a	—
Moderate BPD *Survivors (n* = *63)*	8 (35%)	15 (38%)	NS
*Non- Survivors (n* = *30)*	n/a	n/a	—
Severe BPD *Survivors (n* = *63)*	12 (52%)	17 (43%)	NS
*Non- Survivors (n* = *30)*	n/a	n/a	—
ROP ≥ Grade 3 *Survivors (n* = *63)*	5 (22%)	7 (18%)	NS
*Non- Survivors (n* = *30)*	0	2 (11%)	NS
IVH ≥ Grade 3 *Survivors (n* = *63)*	1 (4%)	1 (3%)	NS
*Non- Survivors (n* = *30)*	4 (36%)*	7 (37%)*	NS
PVL *Survivors (n* = *63)*	2 (9%)	1 (3%)	NS
*Non- Survivors (n* = *30)*	1 (9%)	1 (5%)	NS

Data presented as median (range) for continuous variables or n (%) for categorical variables. % values calculated from total neonates with data available for given variable. Total number of admission to NICU (93 infants; 63 survivors and 30 non-survivors at time of discharge home). *Denotes significant difference (p < 0.05) between survivors and non-survivors within gestational age. NS denotes non-significance.

### Morbidity at time of discharge

There was no difference in age at discharge between infants discharged directly from NICU or step-down level 2 units and there was no difference in major morbidity between those born at 23- or 24-weeks gestation. ROP requiring laser treatment was seen in 4/23 infants (18%) in the 23-week cohort and 6/40 infants (15%) in the 24-week cohort. 11/23 infants (48%) and 14/40 infants (35%) in the 23-week cohort and 24-week cohort respectively were discharged home with supplementary low-flow oxygen. Twenty out of 23 surviving 23-week gestation infants (87%) and 39/40 surviving 24-week gestation infants (97%) had no major neuroanatomical abnormalities (IVH ≥ 3 or PVL) detected at any time during their clinical course.

### Cause and timing of neonatal death

**Figure 2:**
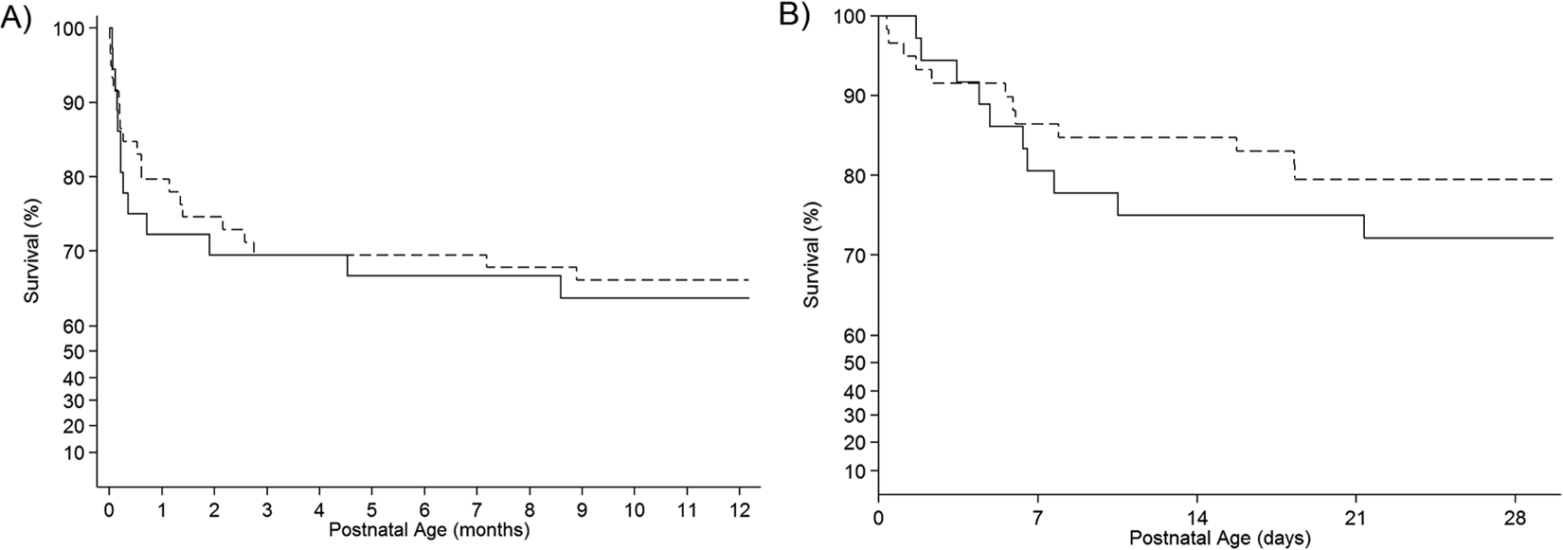
Kaplan-Meier survival estimate curve. Day-by-day actuarial survival rate of periviable infants born at the Wellington Hospital. Data is stratified by gestational age at birth: 23 weeks (solid line) and 24 weeks (dashed line). Only those infants admitted to the neonatal intensive care unit are included. (**A**) Survival to 1-year chronological age (**B**) Enhanced view of survival over first 30 days of life.

The majority of NICU deaths occurred within the first week (Fig. 2). Of the 30 infants who died in NICU, active palliation was instituted in 19 (63%) (Supplementary Table 1). Major morbidity preceding death in infants who were transitioned to palliative care included severe IVH/PVL (n = 6), respiratory failure (n = 3), overwhelming sepsis (n = 1), or a combination of factors (n = 9). For those infants who died whilst still receiving active neonatal care, sepsis and necrotising enterocolitis were the commonest causes of death (Supplementary Table 1).

### Survival at 2 years corrected age

Following discharge from the neonatal units, one 23 weeks gestation infant and four 24 weeks gestation infants died before 2 years corrected age. Overall survival rates of liveborn infants is therefore 58% (22/38) and 60% (36/60) in 23-week and 24-week cohorts respectively at 2 years corrected postnatal age (Fig. 1).

### Health and wellbeing amongst survivors at 2 years corrected age

Of the surviving children at 2 years corrected postnatal age for whom health status data were available (19/22, 86%, in the 23 week cohort; 31/36, 86%, in the 24 week cohort), 11/19 (58%) and 23/31 (74%) were free of any moderate or severe health impairments, in the 23- and 24-week cohorts respectively (Table 4). Health status data were not available for three in the 23-week cohort and five in the 24-week cohort, in two cases due to families moving overseas. As specialist health care for children in New Zealand is publically funded, this suggests that the children lost to medical follow up but domiciled in NZ are unlikely to have major health needs.

**Table 4:** Disability among survivors at 2 years corrected age.

	23 weeks GA (n = 19)*	24 weeks GA (n = 31)*	p-value
**Moderate disability overall**	**4 (21%)**	**5 (16%)**	NS
Motor	3 (16%)	0	
Cognitive	3(16%)	0	
Hearing	1 (5%)	0	
Speech and language	1 (5%)	4 (13%)	
Vision	1 (5%)	2 (6%)	
Respiratory	0	0	
Gastrointestinal	0	1 (3%)	
Renal	0	0	
**Severe disability overall**	**4 (21%)**	**3 (10%)**	NS
Motor	1 (5%)	0	
Cognitive	3 (16%)	2 (6%)	
Hearing	0	0	
Speech and language	1 (5%)	1 (3%)	
Vision	0	0	
Respiratory	1 (5%)	0	
Gastrointestinal	1(5%)	0	
Renal	0	0	

Overall diagnosis of moderate or severe disability was given based on the most severe disability found. *Total numbers of survivors at 2 years of age for whom complete medical records are available.

## Discussion

In the current study, we have clearly demonstrated that where management of the periviable infant includes provision for active perinatal care with maternal delivery at a tertiary neonatal centre, survival free of significant disability at 2 years corrected postnatal age can be achieved. In addition, birth at 23 weeks gestation did not confer any additional burden of surgical or medical complexity when compared to infants born at 24 weeks gestation, and survival rates of infants born at 23 weeks gestation were comparable to those of infants born at 24 weeks gestation.

Birth at less than 24 weeks gestation remains uncommon, and consequently the ‘optimal’ perinatal care of infants born prior to 24 weeks gestation remains unclear^12,20–23^. These complexities are reflected in the different gestational age thresholds at which resuscitation and active care is initiated^24,25^. However, in New Zealand and across the world, there is no formally agreed national consensus in practise, leading to variation between tertiary institutions and their referring regional hospitals. Differences in local and international practice make comparisons between centres challenging. Many centres report widely different rates of antenatal corticosteroid use, operative birth and other perinatal care strategies that may explain the variation in morbidity and mortality^20,23,24^. Furthermore, early trimester dating scans have a margin of error that undermines treatment threshold decisions based on gestational age alone^26^. Similarly, the effect of other variables, including corticosteroid exposure can modify survival risk significantly^11^. Thus if an *a priori* decision has been made to withhold corticosteroids at 23 weeks, the infant will never have the benefit of ‘optimised’ care and outcomes will inevitably be less good than they could be. Survival rates may be described relative to the total number of births, the number of infants live born, or the number of infants admitted to the NICU^27^. We suggest for meaningful counselling of parents as well as comparison between centres, survival is reported based on the number of liveborn babies, with those admitted to NICU as well as discharged home reported to avoid any bias with either over or under-reporting.

In the current study, we report survival rates at 2 years corrected age of 58% and 60% amongst all live born infants in the 23- and 24-week gestation cohorts. Our survival rates are comparable with the recently published Japanese and Swedish data^25,28^ although we report lower rates of neurological injury in survivors than the Japanese cohort. In both our cohort and the Swedish cohort, antenatal corticosteroid exposure and operative delivery rates were high. However, in comparison to obstetric practice in Sweden, antenatal dating ultrasound scans in NZ are routine during the first trimester when gestational age may be calculated with a greater degree of precision. Thus, it is possible that Swedish cohorts may include relatively more mature but growth restricted infants^29^. It is also customary in our Unit when infants have developed significant IVH and, or, PVL to discuss possible redirection of care from active intervention to palliation and ‘comfort care’. The striking difference in rates of grade 3/4 IVH between survivors and non-survivors therefore reflects our philosophy of open discussion with parents and wider family, ideally prior to birth, of possible limitation of active care should complications with a significant risk of later neurodevelopmental morbidity develop.

In the current study, BPD and requirement for supplementary low-flow oxygen at discharge were common. However, as lung development continues through childhood, we do not regard this as an indication to exclude 23-week gestation infants from active NICU care. More importantly, we have also shown that most infants discharged home did not have evidence of significant neurological injury (Grade 3/4 IVH: 3/23 (13%) in surviving 23-week gestation infants and 1/40 (3%) in surviving 24-week gestation infants) and rates of moderate to severe disability were comparable between those born at 23 and 24 weeks gestation at 2 years corrected age. A recently published study suggests that a higher rate of survival without impairment in 23 weeks gestation infants is found in those institutions with higher rate of active resuscitation for extremely preterm infants^30^. Our study supports this finding as our high rate of resuscitation in 23 weeks gestation infants did not result in excess morbidity and mortality compared to infants born at 24 weeks gestation.

Our study has a number of limitations that need to be acknowledged. This is a retrospective study where the data derive from a single tertiary centre with materno-fetal medicine and neonatal expertise available on site. The type of resource available to women and their babies is therefore not representative of the majority of birthing centres where access to tertiary services will necessarily be delayed. Similarly, as we wished to describe outcomes where it was perceived that maximal perinatal support was provided we did not include out-born infants requiring postnatal retrieval to tertiary care as this is recognised to significantly increase the risk of morbidity and mortality. Outcomes at 2 years corrected postnatal age are derived as a composite of formal developmental and, or, paediatric assessment. While this is not as robust as a universal standardised health and developmental assessment, it does provide a valuable, holistic overview of the child’s wellbeing while reflecting the limitations of routine clinical follow-up.

## Conclusion

We have shown survival free of moderate or severe disability can be achieved in infants born at 23 weeks gestation when maximal perinatal care is provided in a publically-funded tertiary setting. We also report that, in the current study, there were no clear perinatal factors that reliably predicted infant outcome. Importantly, our high rate of resuscitation in infants born at 23 weeks did not result in a disproportionately high burden of severely neurologically compromised infants. Furthermore, given that most death occurs in the first week after birth our practice of active intervention does not have major adverse implications for resource management. We therefore recommend that dialogue in this area should focus on improving equitable access of high-risk women to maximal perinatal care settings, rather than focus on whether or not to withhold potentially lifesaving treatment in infants born before 24 weeks of gestation.

## Electronic supplementary material

Supplementary Table 1
