# Supplementary material for: Outcomes of 23- and 24-weeks gestation infants in Wellington, New Zealand: A single centre experience
Source: Sci Rep. 2017 Oct 6;7:12769. doi: 10.1038/s41598-017-12911-5 (PMC5630631; doi:10.1038/s41598-017-12911-5)
Supplement: Supplementary file 1 — Supplementary Table 1 [file 41598_2017_12911_MOESM1_ESM.doc]

**Outcomes of 23- and 24-week gestation infants in Wellington, New Zealand: A single centre experience**

Mary Judith Berry, Maria Saito-Benz, Clint Gray, Rebecca Maree Dyson, Paula Dellabarca, Stefan Ebmeier, David Foley, Dawn

Elizabeth Elder, Vaughan Francis Richardson

**Supplementary Table 1. Characteristics of infants who were admitted to NICU and died prior to discharge**

**
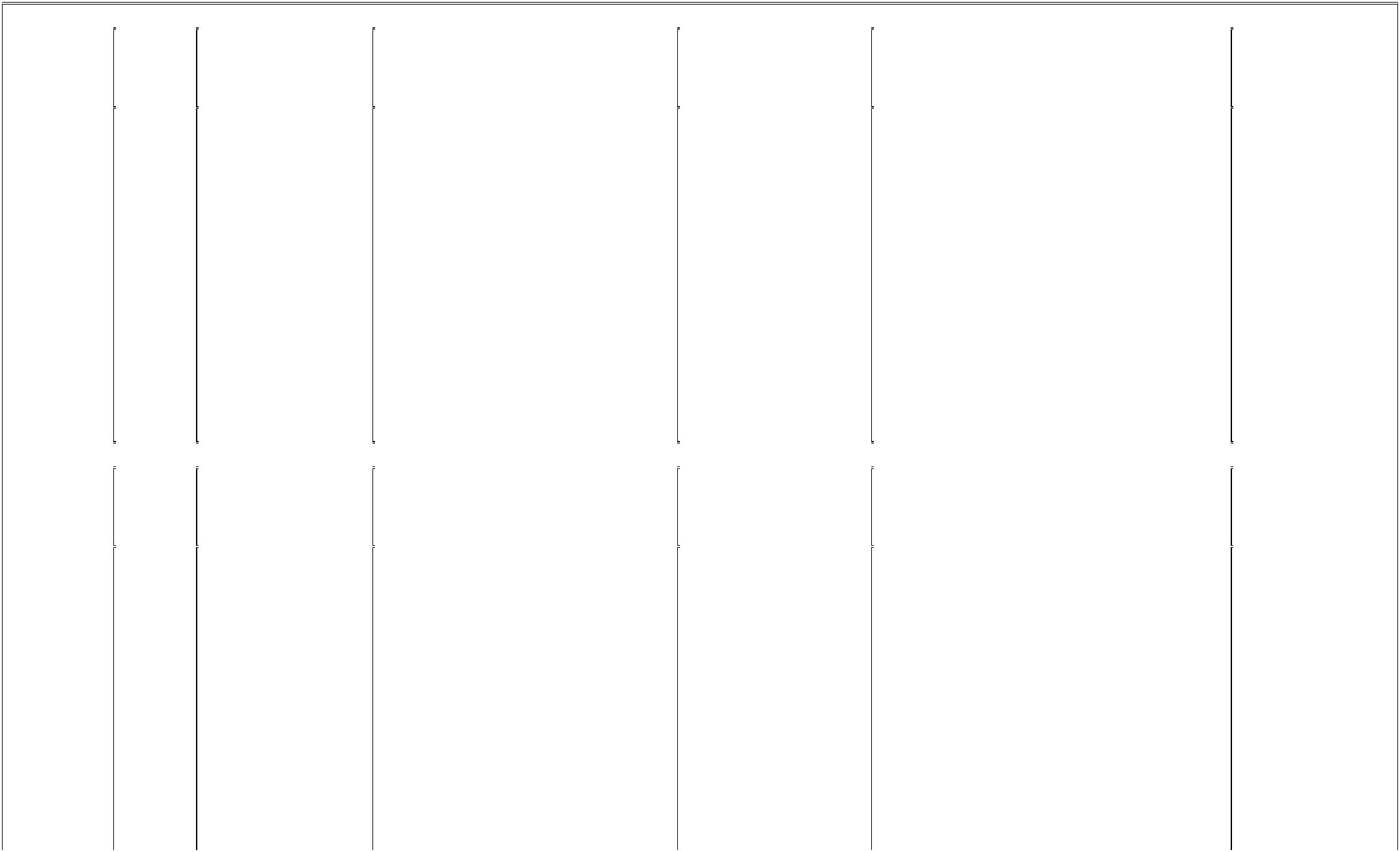
**

| **NICU admission at 23 weeks gestation** | | | | | | |  |  |  |  |  | |  |  |  |  |  |
| --- | --- | --- | --- | --- | --- | --- | --- | --- | --- | --- | --- | --- | --- | --- | --- | --- | --- |
| Birth |  | Sex |  |  | | Antenatal steroids |  |  | Antenatal complications |  |  | | Age at death (days) |  | Cause of death |  | Active palliation |
| weight (g) |  |  |  |  | |  |  |  |  |  |  | |  |  |  |  |  |
|  |  |  |  |  | |  |  |  |  |  |  | | |  |  |  |  |
| 740 |  | M |  |  | | Incomplete |  |  | Antepartum haemorrhage |  |  | | 4 |  | Sepsis |  | Yes |
| 648 |  | F |  |  | | Incomplete |  |  |  |  | 3 | | |  | Gd 3 IVH |  | Yes |
| 621 |  | M |  |  | | Complete |  |  | Antepartum haemorrhage |  | 6 | | |  | PVL, sepsis and meningitis |  | Yes |
| 620 |  | M |  |  | | Complete |  |  | PPROM |  | 257 | | |  | Intractable pulmonary hypertension |  | Yes |
|  |  |  |  |  | |  |  |  | Antepartum haemorrhage |  |  | |  |  |  |  |  |
|  |  |  |  |  | |  |  |  |  |  |  | | |  |  |  |  |
| 651 |  | M |  |  | | Complete |  |  | Maternal congenital cardiac disease |  | 21 | | |  | Gd 4 IVH, meningitis |  | Yes |
| 525 |  | F |  |  | | Incomplete |  |  | Chorioamnionitis |  | 4 | | |  | Gd 4 IVH |  | Yes |
| 615 |  | M |  |  | | Incomplete |  |  | PPROM, chorioamnionitis |  | 57 | | |  | NEC, overwhelming sepsis |  | Yes |
| 635 |  | M |  |  | | Incomplete |  |  |  |  | 3 | | |  | Gd 4 IVH |  | Yes |
| 630 |  | F |  |  | | Complete |  |  | PPROM |  | 6 | | |  | Necrotising pneumonitis |  | No |
| 615 |  | M |  |  | | Complete |  |  | Twin pregnancy |  | 136 | | |  | NEC |  | No |
| 680 |  | M |  |  | | Complete |  |  | Antepartum haemorrhage, PPROM |  | 7 | | |  | Aortic thrombus |  | No |
|  | | | | | | |  |  |  |  |  | |  |  |  |  |  |
| **NICU admis** |  | **sion at 24** |  | | **weeks gestation** | |  |  |  |  |  | |  |  |  |  |  |
| Birth |  | Sex |  |  | | Antenatal steroids |  |  | Antenatal complications |  |  | | Age at death (days) |  | Cause of death |  | Active palliation |
| weight (g) |  |  |  |  | |  |  |  |  |  |  | |  |  |  |  |  |
|  |  |  |  |  | |  |  |  |  |  | | | |  |  |  |  |
| 430 |  | M |  |  | | Complete |  |  | Severe PET |  | | 6 | |  | Respiratory failure |  | Yes |
| 600 |  | M |  |  | | Complete |  |  | Chorioamnionitis |  | 18 | | |  | Gd 3 IVH |  | Yes |
|  |  |  |  |  | |  |  |  | Recreational drug misuse |  |  | |  |  | Overwhelming sepsis |  |  |
| 585 |  | M |  |  | | Complete |  |  | Twin pregnancy, chorioamnionitis |  | 1 | | |  | Gd 4 IVH |  | Yes |
| 655 |  | F |  |  | | Incomplete |  |  | PPROM, chorioamnionitis |  | 1 | | |  | Gd 4 IVH |  | Yes |
| 760 |  | M |  |  | | Complete |  |  | PPROM, chorioamnionitis |  | 64 | | |  | NEC, renal failure |  | Yes |
| 655 |  | F |  |  | | Incomplete |  |  | PPROM, chorioamnionitis |  | 1 | | |  | Gd 4 IVH |  | Yes |
| 690 |  | M |  |  | | Complete |  |  | Twin pregnancy |  | 15 | | |  | Overwhelming sepsis |  | Yes |
|  |  |  |  |  | |  |  |  | PPROM, chorioamnionitis |  |  | |  |  | Respiratory failure |  |  |
|  |  |  |  |  | |  |  |  |  |  |  | |  |  | Intestinal perforation |  |  |
| 675 |  | F |  |  | | Incomplete |  |  |  |  | 18 | | |  | Gd 4 IVH, NEC |  | Yes |
| 550 |  | F |  |  | | Complete |  |  | Severe PET |  | 2 | | |  | Gd 3 IVH, Respiratory failure |  | Yes |
| 665 |  | M |  |  | | Complete |  |  | Antepartum haemorrhage |  | 82 | | |  | Volvulus, NEC |  | Yes |

| 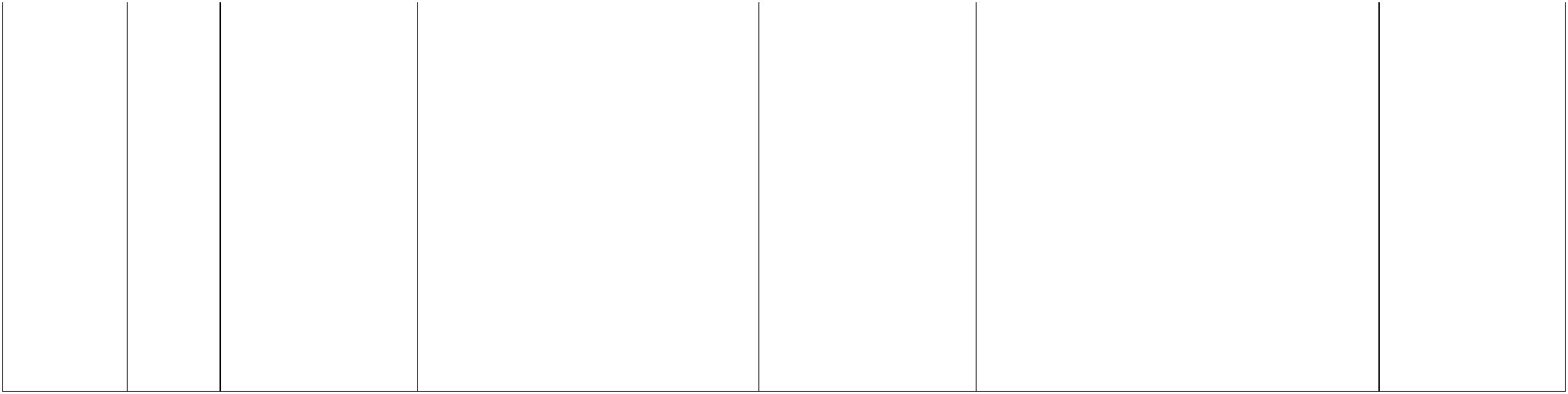 |  |  |  |  | Twin pregnancy |  |  |  |  |
| --- | --- | --- | --- | --- | --- | --- | --- | --- | --- |
|  |  |  |  |  | Chorioamnionitis |  |  |  |  |
| 545 | M |  | Incomplete |  | Grade 4 placental praevia, |  | 215 | Respiratory failure | Yes |
|  |  |  |  |  | Severe PET |  |  | Pulmonary hypertension |  |
| 624 | F |  | None |  |  |  | 40 | Overwhelming sepsis, Gd 4 IVH, PVL | No |
| 538 | F |  | Complete |  | Severe PET |  | 5 | NEC, overwhelming sepsis | No |
| 625 | M |  | Complete |  | Antepartum haemorrhage |  | 24 | Fulminant NEC | No |
|  |  |  |  |  | Twin pregnancy Chorioamnionitis |  |  |  |  |
| 745 | F |  | Complete |  | Chorioamnionitis |  | 1 | Disseminated intravascular coagulation | No |
| 645 | M |  | Complete |  | Recreational drug misuse |  | 146 | NEC | No |
| 615 | M |  | Complete |  | Twin pregnancy |  | 41 | Overwhelming sepsis | No |
|  |  |  |  |  | PPROM, chorioamnionitis |  |  |  |  |
| 630 | M |  | Incomplete |  | Severe PET |  | 78 | Necrotising fasciitis | No |
| 800 | M |  | Complete |  | Twin pregnancy |  | 5 | Overwhelming sepsis | No |
|  |  |  |  |  | PPROM, chorioamnionitis |  |  | Pulmonary haemorrhage |  |

PPROM indicates premature prolonged rupture of membranes. PET indicates preeclamptic toxaemia. IVH indicates intraventricular haemorrhage. PVL indicates periventricular leukomalacia. NEC indicates necrotising enterocolitis.
